# Knowledge and Awareness About Diabetes Mellitus Among Urban and Rural Population Attending a Tertiary Care Hospital in Haryana

**DOI:** 10.7759/cureus.38359

**Published:** 2023-04-30

**Authors:** Dr.Lalit Kumar, Rahul Mittal, Akhil Bhalla, Ashwani Kumar, Hritik Madan, Kushagra Pandhi, Yukta Garg, Kamaldeep Singh, Arpit Jain, Surya Rana

**Affiliations:** 1 Internal Medicine, Adesh Medical College and Hospital, Shahbad, Kurukshetra, IND; 2 Pain Medicine, Adesh Medical College and Hospital, Shahbad, Kurukshetra, IND; 3 Pharmacy, Adesh Institute of Pharmacy and Biomedical Sciences, Adesh University, Bathinda, IND; 4 Internal Medicine, Jawaharlal Nehru Medical College, Chandigarh, IND; 5 Emergency Medicine, All India Institute of Medical Sciences, New Delhi, New Delhi, IND

**Keywords:** obesity, myths, tertiary hospital, rural, urban, prevention, alternative medications, awareness, knowledge, diabetes

## Abstract

Background: Diabetes mellitus (DM) is one of the fastest-growing public health problems in the twenty-first century. The ignorance among people about their disease may be related to their low socioeconomic status and lack of quality education available to them about the disease. It is a serious condition leading to several complications if the individual does not follow up regularly for check-ups and blood sugar monitoring. Lifestyle modifications such as a healthy diet, regular exercise, reducing weight, stress management, and smoking cessation can play a critical role in managing diabetes and improving the health and well-being of diabetic patients. Thus, through this study, we want to assess and create awareness among diabetic patients.

Methodology: It is a hospital-based cross-sectional study conducted at a tertiary care hospital on diagnosed cases of DM. The patients aged 18 years or above of either gender who had already been diagnosed with DM type 1 and type 2 were included, and patients with gestational DM were excluded from the study. Informed consent was taken from the patients, and all the required details were obtained using a well-structured questionnaire. After obtaining all the answers, the level of knowledge and awareness was analyzed, and the data was entered into an MS Excel sheet (Microsoft, Redmond, Washington) and analyzed by Statistical Package for the Social Sciences (SPSS) version 22.0 (IBM Corp., Armonk, NY).

Results: In our study, the maximum prevalence of diabetes was seen in males (55.5%) than females (44.5%), and the mean age of our study population was 53.3 ± 16.4 years. In our study, participants from rural areas made up the majority (59%) compared to those from urban areas (41%), and the majority of participants had a high school education. Among 211 diabetics, about 84%, 79%, and 41% of the patients knew about diabetes, symptoms of diabetes, and complication of diabetes. Only 18% of the patients were aware of the symptoms of hypoglycemia, and 38% of the patients possess their own glucometers and monitor their blood sugar levels on a regular basis. Merely 38% of the diabetics were aware of the various DM treatment choices. About 52% of patients had some awareness of insulin therapy. Out of 211 patients, about half skipped their antidiabetic prescriptions, and of those, 22% took a double dose the next day. A total of 121 patients (57%) combined the use of alternative and allopathic medications, and among these, 22% of patients had replaced the allopathic with alternative medicines. Almost 53% of patients had a positive family history of diabetes; 54% of patients believe that obesity is unrelated to diabetes, and 79% of diabetics are aware of the lifestyle changes that must be done for diabetes. Almost 67% of the patients believed that diabetes could be permanently treated, and 84% of patients believed that eating too much sugar caused their diabetes.

Conclusion: In our study, a significant number of patients suffering from diabetes had less knowledge and awareness about it. The prevalence of myths about the onset of diabetes was noticeably higher among diabetic patients. It was observed that a greater number of patients were shifting to alternative medications instead of allopathic ones, and in the long run, it can lead to various complications. Therefore, there is an immediate need to promote awareness about diabetes among the general population.

## Introduction

Diabetes mellitus (DM) is one of the fastest-growing public health problems in the twenty-first century. According to the International Diabetes Federation (IDF) 10th edition, the prevalence of diabetes is 537 million in 2021, and it will exponentially rise to 643 million by 2030 and 783 million by 2045, out of which 50% of cases (i.e., one in two adults with diabetes) remain undiagnosed [1]. It was seen in a study that India ranks second, while China ranks first in the global diabetes epidemic [2]. India will have a prevalence of about 79.4 million diabetic cases by the year 2030 [3]. Many people (around 45%) remain undiagnosed for years and are later diagnosed as a case of type 2 DM [1].

The ignorance among people about their disease process may be related to their low socioeconomic status and lack of quality education available to them about the disease. Some people come to know about their disease once it has led to various complications like retinopathy, nephropathy, and neuropathy. Therefore, increasing awareness and education of people about the disease may help people to control their blood sugar levels and prevent its complications [4].

Diabetes is a serious condition leading to several complications if the individual does not follow up regularly for check-ups and blood sugar monitoring [5]. There are various treatment options available for diabetes such as insulin injections or insulin pumps in conjunction with continuous glucose monitoring technology for type 1 diabetes and oral hypoglycemic drugs for type 2 diabetes [6]. Lifestyle modifications such as a healthy diet, regular exercise, reducing weight, stress management, and smoking cessation can play a critical role in managing diabetes and improving the health and well-being of diabetic patients [7].

The aim of this study is to create awareness and knowledge about DM and its complications. The objectives of this study are to assess the awareness and knowledge about the disease, its complications, treatment preferences, lifestyle modifications, and self-monitoring of glucose among diabetic patients.

The rationale of this study was that most people in Haryana lack literacy and that diabetes is a significant non-communicable disease that affects a large number of people. It is the need of the hour to raise awareness about diabetes as the incidence of diabetes is increasing nowadays even in the younger age group.

## Materials and methods

It was a hospital-based cross-sectional study on already diagnosed cases of DM. The study was conducted in Adesh Medical College and Hospital, Shahbad, over a period of two months from August 15, 2022, to October 15, 2022, with a sample size of 211 (duration based). The study was conducted after due approval from Institutional Ethics Committee for Biomedical and Health Research (IEC-BHR), Adesh Medical College and Hospital, Shahabad, Haryana, vide reference number AMCH/IEC-BHR/2022/08/01. The patients, aged 18 years or above of either gender, who had already been diagnosed with DM type 1 and type 2 were included, and the patients with gestational DM were excluded from the study.

Patients were included in the study after obtaining informed consent from them. A structured questionnaire was provided to the patients for obtaining demographic details like name, age, sex, occupation, education, and area of residence. It also had basic questions regarding knowledge and awareness of DM. Questions were interpreted by the interviewer to the patients, and forms were filled out according to the answers given by those patients. The form was in English language and interpreted by the interviewer in the local language (Hindi) to those who could not understand or read English. After obtaining all the answers, the level of knowledge and awareness was analyzed. All the data collected were kept confidential. The data were entered into an MS Excel sheet (Microsoft, Redmond, Washington) and analyzed by the Statistical Package for the Social Sciences (SPSS®) version 22.0 (IBM Corp., Armonk, NY). Categorical data were represented as percentages and frequencies. The mean age of diabetes was calculated in terms of mean ± standard deviation.

## Results

Demographic characteristics

The majority of our study sample, 51 patients (24%) who have an average age of 53.3 ± 16.4 years, falls within the age range of 51-60 years as shown in Table 1, while patients above 60 years of age made up 37.4% of the population and patients under 50 years of age made up 38.4%.

**Table 1:** Age distribution

Age	Number of participants	Percentage (%)
18-30 years	25	11.8
31-40 years	28	13.3
41-50 years	28	13.3
51-60 years	51	24.2
61-70 years	46	21.8
71-80 years	33	15.6
Total	211	100.0

In our study, the maximum prevalence of diabetes was seen in males (55.5%) than in females (44.5%) as shown in underlying Table 2. As a result, males are more susceptible to disease than females.

**Table 2:** Gender

Gender	Number of participants	Percentage (%)
Female	94	44.5
Male	117	55.5
Total	211	100.0

In our study, participants from rural areas made up the majority (59%) compared to those from urban areas (41%) as shown in Table 3. Therefore, the rural population has more inclination toward DM than the urban population.

**Table 3:** Area of residence

Residence	Number of participants	Percentage (%)
Rural	124	58.8
Urban	87	41.2
Total	211	100.0

About 32% of patients, or the majority, had high school education; 27% of the patients completed middle school, whereas one-fourth (25%) of our study population was illiterate as shown in Table 4. Only 16% were educated to the graduation level.

**Table 4:** Education

Education	Number of participants	Percentage (%)
Illiterate	53	25.1
Middle school	57	27.1
High school	67	31.7
Graduate	34	16.1
Total	211	100.0

About 84% of the 211 diabetic individuals were aware that having diabetes indicated having higher blood sugar levels as shown in Table 5, and 79% of patients were aware of the symptoms of DM, including frequent urination, increased thirst, persistent hunger, and unexpected weight loss. About 41% of patients were aware of diabetes-related complications, which include blurred vision, nerve damage, renal damage, and various issues with foot and oral health. Only 18% of the patients were aware of the symptoms of hypoglycemia.

**Table 5:** Knowledge and awareness about the disease DM: Diabetes mellitus.

Questions	No	Yes
Knowledge about diabetes	33 (15.64%)	178 (84.36%)
Symptoms of DM	44 (20.86%)	167 (79.14%)
Symptoms of hypoglycemia	172 (81.52%)	39 (18.48%)
Complications of diabetes	124 (58.76%)	87 (41.24%)

Only 38% of the 211 patients possess their own glucometers and monitor their blood sugar levels on a regular basis, yet Table 6 reveals that 57% of those patients are unaware of typical fasting and postprandial blood sugar levels.

**Table 6:** Knowledge and awareness about the blood test of diabetes

Questions	No	Yes
Own a glucometer and check your blood glucose level on a regular basis	130 (61.61%)	81 (38.39%)
Knowledge about normal fasting blood sugar levels and postprandial blood sugar levels	120 (56.87%)	91 (43.13%)

Merely 38% of the 211 diabetics were aware of the various DM treatment choices and duration of treatment as indicated in Table 7. About 52% of the patients knew something about the insulin injections available for the treatment of diabetes.

**Table 7:** Knowledge and awareness about the treatment of diabetes

Questions	No	Yes
Awareness about the various treatment options for diabetes and the duration of treatment	130 (61.61%)	81 (38.39%)
Awareness about the insulin therapy	101 (47.86%)	110 (52.14%)

Out of 211 patients, approximately half (49%) skipped their antidiabetic prescriptions, and of those, approximately half (22%) took a double dose the next day. About 121 patients (57%) combined the use of alternative medications along with allopathic medications, and of these 121 patients, 26 patients (22%) had replaced the allopathic medications with alternative medicines as indicated in Table 8.

**Table 8:** Knowledge and awareness about the medication for diabetes

Questions	No	Yes
Missed your antidiabetic medications	108 (51.2%)	103 (48.8%)
if missed, take a double dose on the next day	80 (77.7%)	23 (22.3%)
Use alternative medications along with allopathic medications	90 (42.65%)	121 (57.35%)
Replaced the allopathic medicines with alternative medicines completely	95 (78.51%)	26 (21.49%)

As shown in Table 9, almost 53% of patients had a positive family history of diabetes. About 54% of patients think that obesity and diabetes are unrelated, and although 79% of diabetics are aware of the lifestyle adjustments required to control their illness, they have not been able to incorporate these changes into their daily routines.

**Table 9:** Risk factors of diabetes DM: Diabetes mellitus.

Questions	No	Yes
Family history of DM	100 (47.4%)	111 (52.60%)
Association with obesity	114 (54%)	97 (46%)
Lifestyle modifications (exercise, dietary changes, smoking, alcohol cessation, etc.)	45 (21.4%)	166 (78.6%)

The majority of patients, nearly 67%, thought that diabetes could be cured permanently. Table 10 shows that 84% of patients thought that having diabetes was due to eating too much sugar.

**Table 10:** Knowledge and awareness about the myths related to diabetes

Questions	No	Yes
Diabetes can be cured permanently	70 (33.18%)	141 (66.82%)
Diabetes is caused by eating too much sugar	35 (16.59%)	176 (83.41%)

## Discussion

DM, simply called diabetes, is a serious, long-term (or “chronic”) condition that is characterized by raised blood glucose levels because the body cannot produce any or enough of the hormone insulin or cannot effectively use the insulin it produces [1]. The typical symptoms are excessive thirst, frequent urination, lack of energy or fatigue, constant hunger, sudden weight loss, and blurred vision [1]. In our study, around 79% of the patients knew these symptoms of diabetes, and about 84% of patients answered that diabetes means raised blood glucose levels. The mean age of our study population was 53 ± 16 years, and the maximum number of patients in the age interval of 51-60 years is 24%. Age is one of the risk factors for the development of diabetes, and age ≥ 60 years is an independent risk factor for diabetes-related complications despite good control of cardiovascular risk factors [8]. In our study, 79 patients (37%) were aged above 60 years. Therefore, diabetes is more prevalent among the elderly, and similar data is seen in previous studies [9,10]. Another key risk factor for DM is a positive family history of diabetes. It is crucial to understand that people with a family history of diabetes are more knowledgeable about the signs and symptoms of the disease as well as the organs impacted by it than people without such a history. In a study conducted in 2017, it was found that in contrast to persons with negative family histories of DM, they experienced early-onset diabetes and were more likely to experience complications [11]. In our study, 53% of individuals were found to have a positive family history of DM out of which 26.4% of patients had knowledge and awareness about diabetes.

Obesity, particularly central obesity, is significantly linked to the onset and progression of type 2 diabetes. In a prior study, patients with type 2 DM had rates of overall obesity, abdominal obesity, and central obesity of 58.68%, 81.84%, and 53.42%, respectively [12]. About 46% of participants in our study were aware of the link between diabetes and obesity. As insulin sensitivity declines in fatty tissues, regulation of beta cell function also declines [13,14]. Increased levels of nonesterified fatty acids (NEFA), glycerol, hormones, cytokines, and pro-inflammatory chemicals in an obese person cause the development of insulin resistance [13].

The diagnosis of DM is made according to the criteria given by the American Diabetes Association (ADA) [15]. A 2020 meta-analysis by Dessie et al. found that a glucometer is significantly associated with higher medication adherence [16]. In our study, only 38% of the patients own a glucometer of their own and keep a check on their blood glucose levels on a regular basis, and 57% of patients do not even know about normal fasting and postprandial blood sugar levels.

In order to treat type 1 diabetes, the traditional method involves checking blood glucose levels manually, followed by daily subcutaneous insulin injections [17]. It has been demonstrated that using insulin pumps in conjunction with continuous glucose monitoring (CGM) technology lowers the long-term risks of diabetic complications [6]. Gene therapy is a novel approach to treating the disease, which offers a promising substitute for insulin injections since it tries to repair defective genes responsible for disease progression and thus prevents or reverses the development of the disease [17,18]. Particularly for type 1 DM, stem cell-based therapy has been viewed as a promising possible therapeutic approach for the management of diabetes [17,19]. For the treatment of type 2 DM, a variety of non-insulin-based oral treatments are available, including biguanides, insulin secretagogues, SGLT2 inhibitors, insulin sensitizers, etc. [20]. In our study, among 211 diabetics, only 38% of the patients were aware of the various treatment options for DM and duration of treatment, whereas about 52% of the patients had some awareness and knowledge about insulin therapy.

A study conducted by Benil et al. in 2003 showed that if a patient forgets to take the prior dose, almost 10% of patients had taken a double dose the next day. However, in our study, about half of the patients missed their antidiabetic medications out of which about 22% of them had taken a double dose on the next day. Also, 57% of patients used alternative medications along with allopathic medications, and 22% of them had replaced allopathic medications completely with alternative medications [21].

Human pluripotent stem cells are an appealing alternative beta cell source for transplantation. Beta cell replacement through islet of Langerhans transplantation is a potential treatment for DM, but the lack of donors prevents its widespread use [22,23]. The benefit upon transplantation is sometimes insufficient, and the transcript causes the functional ability to fall to 60% at 12 months [23,24]. Another suitable limitation that restricts its use is its pocket-draining expenses. After transplantation, the persistence of autoimmunity leads to a lifelong need for immunosuppression, which is a major drawback.

The most significant risk factors for the development of diabetes complications appear to be poor glycemic control and a protracted illness [25]. Diabetes complications affect almost every organ system. Diabetes complications fall into two categories, namely, microvascular and macrovascular. Microvascular issues include diabetic nephropathy, neuropathy, and retinopathy, whereas macrovascular complications include coronary artery disease, stroke, and peripheral vascular disease (PVD) leading to bruises and injuries which do not heal leading to gangrene and ultimately amputation [5]. One of the main barriers to restoring adequate metabolic control of the disease is hypoglycemia, which is the most prevalent acute complication in type 1 diabetes patients. In our study, about 41% of the patients were aware of the complications of DM. Only 18% of the patients were aware of the symptoms of hypoglycemia, i.e., fainting, tremors, convulsions, excessive hunger, etc.

A total of 3,200 participants in the National Diabetes Prevention Program (NDPP) of the United States were randomized to receive standard medical care, metformin therapy, or comprehensive lifestyle intervention. In order to attain a mean objective of 7% weight loss, the lifestyle intervention concentrated on reducing caloric intake by reducing fat calories and increasing physical activity to a goal of at least 150 min/per week [26]. The incidence of type 2 diabetes was shown to have decreased by 58% in the lifestyle intervention group, 31% in the metformin group, and 17% in the routine care group after an average of 2.8 years; thus, lifestyle modifications, i.e., exercise, dietary modifications, and smoking and alcohol cessation, play a major preventable therapy to reduce the disease burden [27]. In our study, around 22% of patients were still unaware of the beneficial and preventive effects of lifestyle modifications.

The majority of the patients believed the misconception that eating more sugar caused diabetes. In our study, 67% of people believe that diabetes can be cured permanently. Other beliefs about diabetes include the notions that it only develops in old age, that bathing your feet in water will help you manage your blood sugar, and that it is caused by your previous misdeeds and can only be treated spiritually [28]. Our study did not ask for any such specific beliefs; hence, there is no data for the same.

There could be several limitations of this study, which could have affected the final outcome. First, the study had highly heterogenous data in regard to the literacy, economic standards, and level of education of the participants involved. The fact that both type I and type II DM were included in the selection process could have a certain effect on the results. There is a possibility of reporting bias as this study was more focused on the knowledge and awareness among participants and not involved in an intervention-based approach. Although these limitations might have some indirect effect on the results, albeit having an insignificant effect on the objectives and final outcome of this study.

## Conclusions

In our study, a significant number of patients suffering from diabetes had less knowledge and awareness about diabetes. The prevalence of myths like diabetes will be permanently cured after taking medications for some time and eating too much sugar causes diabetes was noticeably higher among diabetic patients. It was observed that a greater number of patients were shifting to alternative medications instead of allopathic ones, and in the long run, it can lead to more severe microvascular and macrovascular complications. There is an immediate need to promote awareness about diabetes among the general population with the help of community-based campaigns, collaborations with local media outlets, partnerships with healthcare providers, and optimal use of social media platforms. The stress should be made on early diagnosis and proper management, which are keys to preventing or delaying complications and improving the quality of life for patients with diabetes.

## Appendices

Questionnaire

Name:                                                              Age/Sex:

Education:                                                       Occupation:

Area of Residence: Rural/Urban

**Table 11:** Questionnaire

S. No	Question	Yes/No	Remarks
1.	Do you know about diabetes?		
2	Are you aware of the symptoms of DM?		
3	Are you aware of the symptoms of hypoglycemia?		
4	Are you aware of the various treatment options for DM and the duration of treatment?		
5	Are you aware of insulin therapy?		
6	Do you own a glucometer of your own and do you check your blood glucose level on a regular basis?		
7	Do you know about normal fasting blood sugar levels and postprandial blood sugar levels?		
8	Have you ever missed your antidiabetic medications? If yes, do you take a double dose of medication the next day?		
9	Are you aware of the complications of diabetes?		
10	Are you aware of lifestyle modifications (exercise, dietary modifications, smoking, alcohol cessation, etc.)?		
11	Is there any family history of DM?		
12	Do you use alternative medications along with allopathic medications? If yes, have you ever completely replaced allopathic medicines with alternative medicines?		
13	Do you think diabetes is associated with obesity?		
14	Do you think diabetes can be cured permanently?		
15	Do you think diabetes is caused by eating too much sugar?		
